# Synthesis, characterization, antimicrobial activity, and toxicity evaluation of aminolevulinic acid–silver and silver–iron nanoparticles for potential applications in agriculture

**DOI:** 10.1039/d2ra05135d

**Published:** 2022-10-21

**Authors:** Marcia Regina Franzolin, Isabela Santos Lopes, Daniella dos Santos Courrol, Susana de Souza Barreto, Lilia Coronato Courrol

**Affiliations:** Laboratório de Bacteriologia, Instituto Butantan São Paulo Brazil; Instituto de Ciências Ambientais, Químicas e Farmacêuticas, Departamento de Física, Universidade Federal de São Paulo Diadema São Paulo Brazil lcourrol@unifesp.br

## Abstract

Aminolevulinic acid (ALA) is considered one of the most critical plants growth regulators and essential precursors for chlorophyll biosynthesis; besides, its photodynamic activity can be used to exterminate larvae and microorganisms in plants and soil. Silver nanoparticles (AgNPs) have unique physicochemical properties and potent antimicrobial, antiviral, and antifungal activities, and in agriculture, their application as nanopesticides has been proposed. In this study, silver and silver–iron nanoparticles capped/stabilized with aminolevulinic acid (ALAAgNPs and ALAAgFeNPs) were synthesized by the photoreduction method and characterized by UV-vis spectroscopy, transmission electron microscopy, and zeta potential analysis. The kinetics of ^1^O_2_ generation from ALAAgFeNPs were obtained. The ALANP toxicity was evaluated on stalks of *E. densa* by observing cell morphology changes and measuring chlorophyll content compared with water-treated plants. Antimicrobial activity was tested against *E. coli*, *P. aeruginosa*, and *Candida albicans*. The results suggested that ALANPs (prepared with [AgNO_3_] ≤ 0.2 mM and [ALA] ≤ 0.4 mM) could be suitable for applications in the agricultural sector. The presence of ∼0.3 mmol of iron in ALAAgNPs synthesis increased cell uptake and chlorophyll synthesis.

## Abstract 2

ALA is a natural metabolite in all living cells and possesses low toxicity. ALANPs exhibit high antimicrobial activity, promote plant growth and have the potential to show photodynamic herbicidal properties under solar illumination.

## Graphical abstract

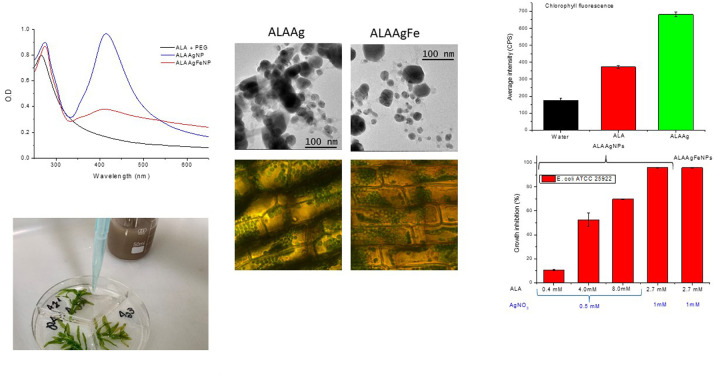

## Introduction

Efforts to increase food production are a significant global challenge, considering population growth, limited terrestrial and water resources, climate change, and high incidence of diseases and pests.^1^ The bioaccumulation of agrochemicals with toxic or carcinogenic compounds and subsequent contamination of the food chain and the environment has fomented a green revolution. New solutions have been proposed as biofertilizers, biopesticides, and biostimulants.^2,3^

Aminolevulinic acid (ALA) is a precursor of tetrapyrrole compounds, such as chlorophyll, playing a vital function in plant photosynthesis and cellular energy metabolism.^4–6^ ALA was first used in agriculture as a biodegradable herbicide and plant growth-promoting factor.^3,7^ ALA has been used as a safe, environmentally compatible, and biodegradable novel plant growth regulator.^8–10^ Furthermore, it was used as a novel pesticide in agriculture.^11^

ALA, one of the second generations of photosensitizers, has been widely applied in photodynamic therapy.^12,13^ Exogenous application of ALA on yeast, insects, and plants induced a high accumulation of protoporphyrin IX (PpIX). PpIX is a generator of reactive oxygen species (ROS), including superoxide, hydrogen peroxide, hydroxyl radical, and singlet, oxygen when excited by light, at an appropriate wavelength, and in the presence of oxygen.^14^ ROS promotes cellular toxicity effects through apoptosis, necrosis, and autophagy. Studies demonstrated that ALA-PDT could be improved by adding iron chelators making ALA-PDT more efficient.^15^ ALA-PDT effectiveness can also be enhanced by combining ALA with nanoparticles.^16–20^ PDT efficacy in the presence of combined ALA and silver nanoparticles (ALAAgNPs) was demonstrated, resulting in higher efficiency than ALA alone.^20,21^

AgNPs can be synthesized by several physical, chemical, and biological methods, and engineered nanomaterials^22–27^ have various biomedical, food processing, and agrochemical applications when conjugated with a ligand. Silver nanoparticles (AgNPs) are an effective pest management agent in agriculture, on account of known surface plasmon resonance (SPR) characteristics, and are a potential tool to act as nanopesticides.^28–32^ Nanosilver has an effective antifungal and antimicrobial activity due to the broad-spectrum and varied modes of action for living organisms.^22,33,34^ It has been used as a potential candidate to increase crop yield by enhancing seed germination and plant growth.^28^

Indeed there are ecotoxicological concerns about the deliberate use of nanoparticles in the agricultural ecosystem since they can affect environments such as soil, water, and plant systems, eventually affecting consumers.^35–37^ The physicochemical properties of nanoparticles, size, surface coating, concentration, shape, agglomeration state, and dose determine the nanotoxicology.^38,39^ The toxicity is induced by decreased chlorophyll content, viable cell counts, and increased ROS generation.^40^

The main goal of this study is to evaluate the potential use of silver (ALAAgNPs) and bimetallic silver-iron (ALAAgFeNPs) nanoparticles surrounded/capped and stabilized by ALA in agriculture. For this, ALANPs were synthesized by the photoreduction method and characterized by UV-vis, TEM, and zeta potential. The release of singlet oxygen by ALAAgFeNPs under excitation around 590 nm was investigated. To evaluate ALANPs toxicity was chosen the *Egeria densa* Planch (*E. densa*), an aquatic, freshwater perennial plant of the family Hydrocharitaceae native to South America, specifically Brazil, Uruguay, and Argentina.^39,41,42^ The fluorescence of chlorophyll extracted from *E. densa* incubated with ALANPs with different silver and ALA concentrations was studied. Furthermore, the antimicrobial activity of synthesized nanoparticles was evaluated through an *in vitro* nanoparticle investigation.

## Materials and methods

### ALAAgNPs synthesis

Silver nitrate and 5-aminolevulinic acid hydrochloride ∼98% (A3785) and polyethylene glycol 10 000 (PEG) were purchased from Sigma-Aldrich. ALA silver nanoparticles (ALAAgNPs) solutions were prepared by mixing silver nitrate with ALA in distilled water at 25 °C. PEG (30 mg) was added to the solutions to improve NPs stability.^43,44^ The process was accompanied by vigorous stirring for 1 minute, and the resulting transparent solution was exposed to a 300 Watts Cermax xenon lamp for 2 minutes. The Xe lamp was positioned at 10 cm of the solution container. The illuminated region covered exactly the recipient diameter, and the intensity of the solution was estimated to be 3.6 W cm^−2^. After illumination, the solution color turned grayish brown.

Hybrid particles (silver–iron) were prepared by adding the iron powder (Brasil 3B Scientific) to distilled water. Then the ALA and AgNO_3_ were added to the solution and illuminated for 2 min.

The AgNO_3_, ALA, and iron powder concentrations used in ALANPs synthesis are shown in Table 1. All nanoparticles had the pH adjusted to 7.0 after irradiation.

**Table 1:** AgNO_3_, iron powder and ALA concentrations used to prepare ALANPs

ALA solution and ALANPs	AgNO_3_ (mM)	Iron (mmol)	ALA (mM)
ALA1		—	2.7
ALAAg2	1	—	2.7
ALAAgFe3	1	1	13.5
ALAAg4	0.2	—	**2.7**
ALAAg5	0.4	—	**2.7**
ALAAg6	1.1	—	**2.7**
ALAAg7	**0.5**	—	0.4
ALAAg8	**0.5**	—	4.0
ALAAg9	**0.5**	—	8.4
ALAAgFe10	0.5	0.3	3.2

### ALANPs characterization

The UV-vis absorption spectra were measured by UV-vis Shimatzu MultiSpec 1501 spectrophotometer.

The stability of the colloidal suspensions was analyzed by zeta potential and dynamic light scattering (DLS) using the Zetasizer Nano ZS Malvern apparatus.

Transmission Electron Microscopy (TEM) images of prepared nanoparticles were obtained. The samples were dripped onto a copper grid and analyzed on JEM 2100 – JEOL transmission electron microscope.

### Plant material

Stalks of *Egeria densa* Planch. (12 cm in length) were grown in test tubes filled with tap water. The temperature was kept at 22 ± 2 °C with day/night light conditions. The water was changed every three days.

### Treatments and sampling

**Fig. 1:**
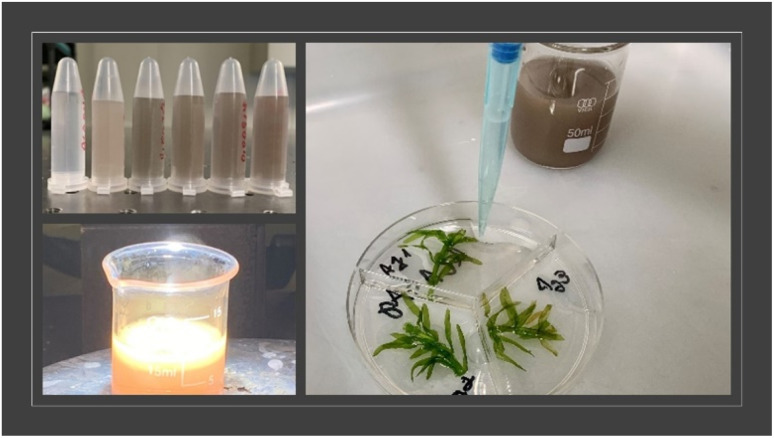
ALAAgNPs (4–9), synthesis during photoreduction (down image), and *E. densa* treatment.

*E. densa* stalks were cut, weighed (0.10 ± 0.03 g), and placed in Petri dishes with 90 mm with three compartments containing 5 mL of distilled water distributed in groups: control group: incubated with 1 mL of water, ALA group: incubated with 1 mL of ALA, and ALAANPS groups: incubated with of ALAAg and ALAAgFeNPs. The dishes were kept under day/night conditions at 22 ± 2 °C for 24 and 72 hours. Fig. 1 exemplifies the experiments. All experiments were performed in triplicate. The concentration of nanosilver was estimated at around 200 nM.

### Plant characterization

To determine the chlorophyll content, *E. densa* treated with water, ALA and ALANPs were washed with distilled water and added to tubes containing 5 mL of acetone to extract chlorophyll. These solutions were centrifuged for 15 minutes at 4000 rpm, and the supernatants were analyzed on an RF-5301 PC Shimatzu fluorimeter. The chlorophyll fluorescence spectra were obtained under excitation at 431 nm, and emission spectra were measured between 550 and 800 nm. An average spectrum was obtained from the signals of 3 samples of each group.

After ALANPs incubation time, *E. densa* leaves images were obtained with Leica DMI6000 CS fluorescence microscope in TL-BF mode, and light intensity, detector gain, and acquisition time were fixed for comparisons. Images were obtained by a color DFC450FX digital video Camera and analyzed by Leica AF6000 software.

The scanning electron microscopy (SEM) images and elemental and material analysis (energy-dispersive X-ray spectroscopy – EDS) of leaves incubated with ALANPs were obtained with a JSM-7610F JEOL microscope. In this case, after NPs incubation, leaves were fixed onto conductive carbon glue and recovered with gold film.

### Indirect release of singlet oxygen

A solution containing 10 μL of 1,3-diphenylisobenzofuran (DPBF) dissolved in DMSO [4.0 mmol L^−1^], and 1 mL of ALAAgFeNPs was prepared. The solution was placed into a 10 mm path-length cuvette and irradiated with 590 ± 10 nm (InGan), MM Optics Venus Sigma LED, with an incident power of 110 mW (fluence rate ∼37 mW cm^−2^). Irradiation was stopped at regular intervals (1 min), and the absorbance spectrum was recorded (Shimatzu MultiSpec 1501).

The increase in the concentration of ^1^O_2_ over time ([^1^O_2_]_*t*_) was calculated from the decrease in DPBF concentration [DPBF]_0_ − [DPBF]_*t*_, according to Beer's law:

1 *A* = *ε* × *b* × *c*

*A*_0_ and *A*_*t*_ are the solution absorption at 422 nm at the beginning and in the measurement's time “*t*”. The quantity of ^1^O_2_ generated by irradiation was calculated according to (eqn (2)).

2 [^1^O_2_]_*t*_ = [DPBF]_0_ − [DPBF]_*t*_ = (*A*_0_ /*ε*_DPBF_) − (*A*_*t*_ /*ε*_DPBF_)

where [DPBF]_0_ and [DPBF]_*t*_ represent the concentrations of DPBF at the beginning of the reaction and the time “*t*” respectively, and *ε*_DPBF_ is the molar absorption coefficient of DPBF around 422 nm (*ε*_DPBF_ = 2375 L mol^−1^ cm^−1^).

### Antibacterial efficacy of ALAAgNPs and ALAAgFeNPs

The microorganisms employed in this assay were: *Escherichia coli* ATCC 25922, *Pseudomonas aeruginosa* ATCC 27853, and *Candida albicans* ATCC 10231.

The antimicrobial activity of ALAAgNP (#2, 7, 8, and 9) and ALAAgFeNP10 nanoparticles was tested in triplicate in 96-well microtiter plates, according to the CLSI guidelines (CLSI, 2015). Aliquots of 50 μL of Mueller–Hinton (MH) broth (bacteria) or Sabouraud (Sab) broth (*C. albicans*), with bacterial or fungal inoculum adjusted to approximately 10^6^ CFU per mL, were incubated with 50 μL of ALANPs in MH or Sab broth. After 20 hours of incubation at 37 °C, microbial growth was measured by observing the optical density (OD) changes at 595 nm in an enzyme-linked immunosorbent assay (ELISA) reader (Multiskan®EX – Thermo Fisher Scientific, EUA). The results were stated as inhibition percentage of OD against a control (microorganisms in the absence of NPs). The following formula calculated the rate of microbial inhibition:
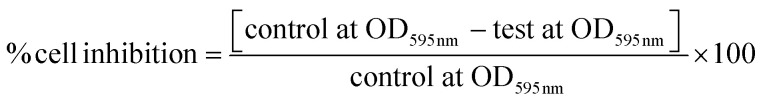


The minimum inhibitory concentration of ALA for dark toxicity is 62.5 μM for *E. coli*.^45^ For *C. albicans*, 15 mM of ALA promotes ∼13% cell death.^46^

### Statistical analysis

All studies were performed in triplicate. The results were statistically compared (Two-sample *t*-test, using *T* distribution) to controls.

## Results

### Nanoparticle's characterization

**Fig. 2:**
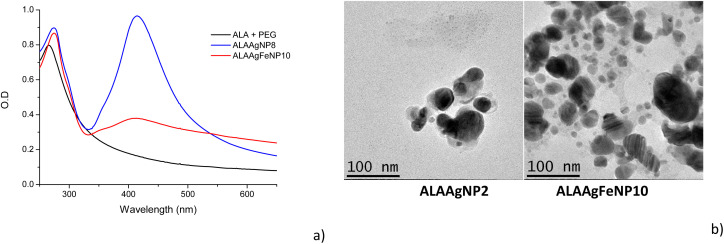
(a) UV-vis spectra of ALA, ALAAgNP8 and ALAAgFeNP10. (b) TEM image of the samples: ALAAgNP2 and ALAAgFeNP10.

The absorbance spectra of the ALA1, ALAAgNP8, and ALAAgFeNP10 solutions are shown in Fig. 2a. Aminolevulinic acid exhibits the characteristic absorbance peak at 267 nm. The ALAAgNP8 solution presented the SPR (surface plasmon resonance) peak at 415 nm, indicating the presence of silver nanoparticles. AgFeNP10 gives broadband beginning around 350 nm.

The transmission electron microscopy (TEM) shown in Fig. 2b indicated nanoparticles with spherical shapes. It is possible to observe that ALAAgFeNP10 presented two-sized nanoparticles, where larger particles were attributed to silver and the smallest to iron (∼30 nm AgNPs and ∼12 nm Fe NPs).

The zeta potential was predominantly negative at −36.6 ± 8.45 mV and −30.8 ± 4.40 mV for ALAAgNP and ALAAgFeNP, respectively, indicating that silver and silver iron nanoparticles present high stabilities. The polydispersity index for nanoparticles was 0.319 and 0.336 for AgNPs and AgFeNPs, respectively.

### Indirect release of singlet oxygen

The indirect release of singlet oxygen was studied using the DPBF sensor for ALAAgFeNPs with a large absorption band in the wavelength next to the solar radiation spectrum (Fig. 2a).

**Fig. 3:**
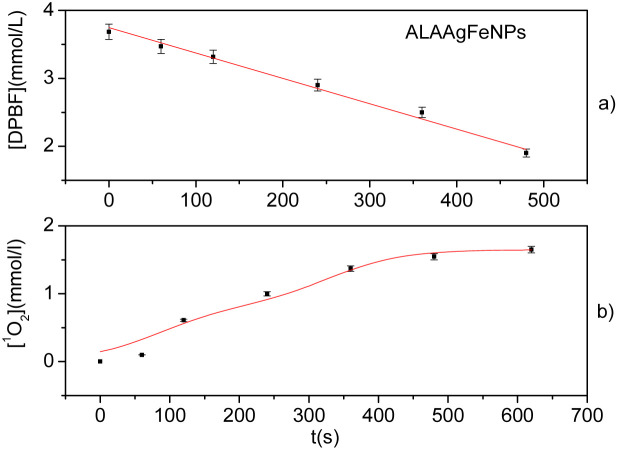
(a) DPBF consumption by irradiation with LED (590 ± 10 nm) of ALAAgFeNPs. DoseResp fit. (b) The quantity of ^1^O_2_ generated by irradiation in the function of the time for ALAAgFeNps. BiDoseResp Fit. The data by UV-vis measurements and performed in triplicate.

The DPBF has a characteristic absorbance band at 422 nm. This band decreases in the presence of singlet oxygen (^1^O_2_).^47^Fig. 3a shows the photodegradation of DPBF in the presence of ALAAgFeNPs in DMSO by irradiation with LED (∼590 ± 10 nm). The reaction was monitored with different exposure times. Fig. 3b shows the calculated concentration generated by ^1^O_2_ in the millimolar range.

### Nanoparticles in *E. densa*

**Fig. 4:**
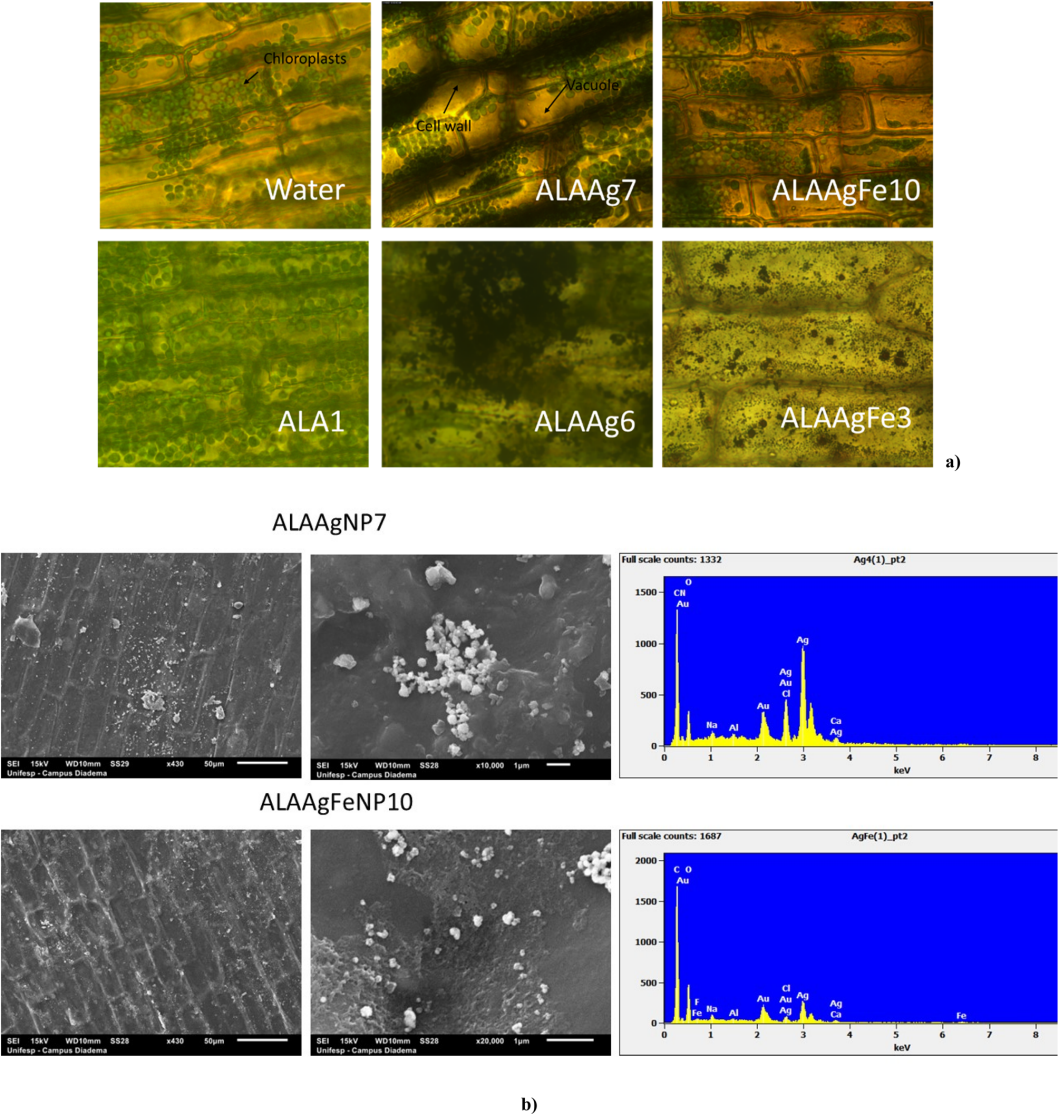
(a) Optical images of *E. densa* treated with water, ALA1, ALAAgNP7, ALAAgFeNP10, ALAAgNP6, ALAAgFeNP3 (objective 100×). (b) Scanning electron microscopy images for ALAAgNP7 and ALAAgFeNP10 (50 μm and 1 μm scales) and energy dispersive spectroscopy (EDS) indicates the presence of silver in leaves.

Fig. 4a shows the images obtained for the leaves of *E. densa* after 72 h incubation with samples from control, ALA, and ALANP Groups. Leaf cell walls and chloroplasts were observed.^42,48^ Plants from the ALA Group presented a higher number of chloroplasts distributed in all cells.

For ALAAgNP7 and ALAAgFeNP10 treated *E. densa*, a high concentration of chloroplasts was observed in the vacuole compressed together due to plasmolysis. The darker color of cell membranes indicates that many ALAAgNP7 were retained in the cell wall. In ALAAgFeNP10-treated plants, red color in the background due to the presence of iron is observed, and the darker color of the vacuole indicates the presence of nanoparticles or silver ions in the cell sap. In the case of nanoparticles with higher silver and iron concentrations (ALAAgNP6 e ALAAgFeNP3, respectively), damage and disruption of plant cell and chloroplast structures were observed. The leaves acquired a gray or red color for ALAAgNP6 and ALAAgFeNP3-treated samples, respectively.

The substantial bioaccumulation of ALANPs by *E. densa* was observed in SEM images shown in Fig. 4b. ALAAgNP7-treated plants presented an agglomerate of silver nanoparticles on the cell surface. In contrast, for ALAAgFeNP10-treated plants, the nanoparticles were well distributed. EDS confirmed the presence of nanoparticles in the leaves.

### Chlorophyll fluorescence

**Fig. 5:**
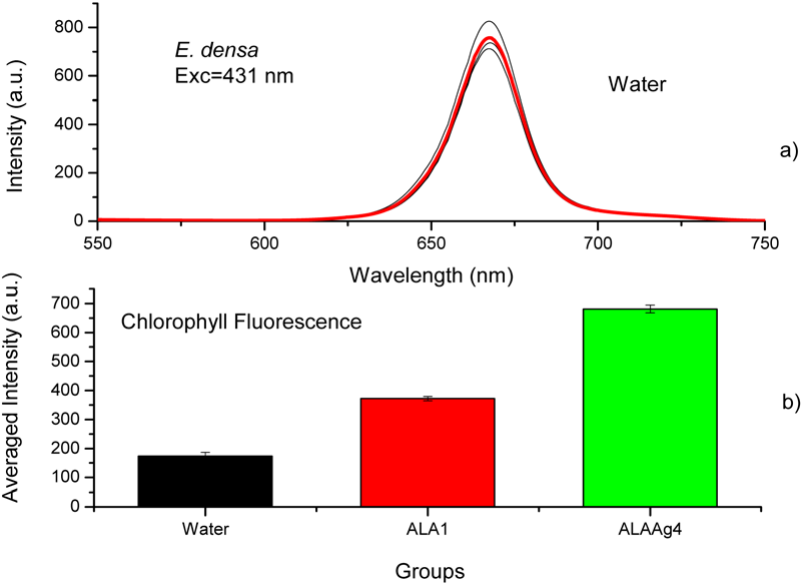
(a) Chlorophyll fluorescence spectra obtained with excitation at 431 nm for samples from the control group (red line – averaged spectrum). (b) Comparison between the averaged chlorophyll fluorescence intensity of plants incubated with water, ALA1, and ALAAgNP4. Two-sample *t*-test, using *T* distribution (two-tailed) between water and ALA groups and between water and ALAAg groups, resulted in a *p*-value <0.001 (0.1%), which is highly significant.

The chlorophyll fluorescence spectra of plants treated with water, ALA, or ALANPs were measured to determine if ALANPs present the same plant-growth-promoting ability as ALA and the toxicity of studied nanoparticles. Fig. 5a shows the fluorescence spectra of chlorophyll extracted from samples of the Control Group (water) obtained with excitation at 431 nm. The average spectrum (red line) shows an emission peak at 668 nm attributed to chlorophyll. Fig. 5b compares the averaged chlorophyll fluorescence intensity obtained for samples from Control, ALA, and ALAAg Groups. The results indicated that the fluorescence intensity for ALA-treated plants was ∼2 times higher than for plants treated with water. In contrast, for ALAAgNPs-treated plants, an increase of ∼ four times was observed. This result indicated that ALA present on the surface of nanoparticles was released inside plants and metabolized to produce chlorophyll. Nanoparticles improved the entrance of ALA in the plant cell.

**Fig. 6:**
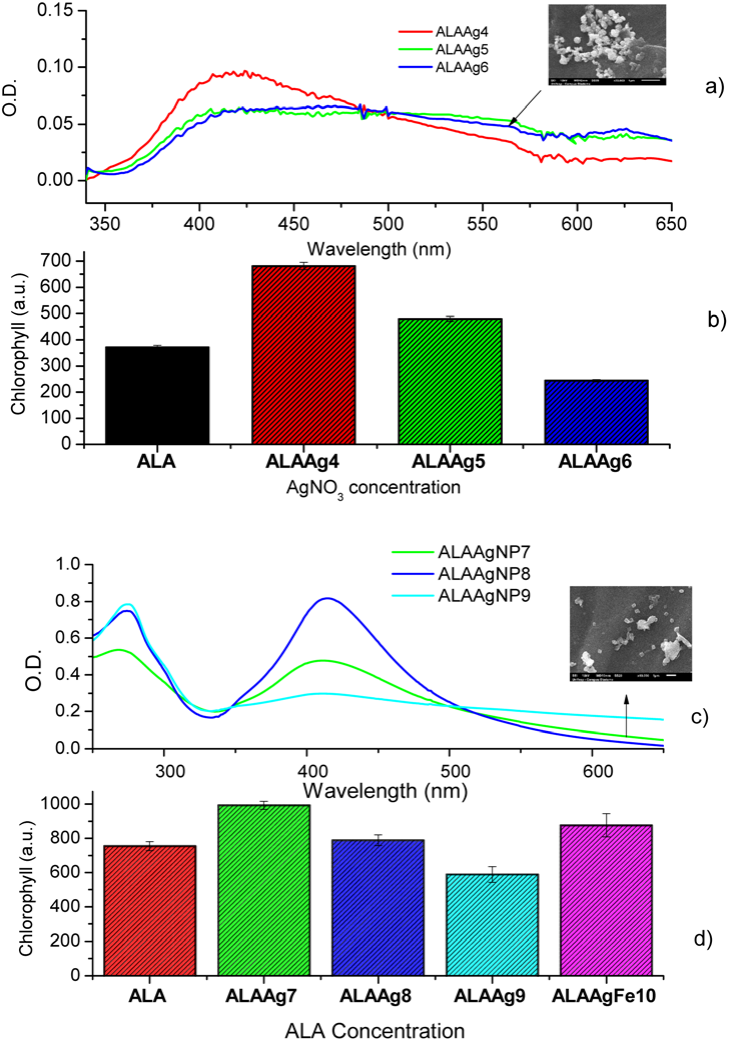
(a) The role of silver concentration in the UV-vis spectra and (b) chlorophyll production. SEM image of a plant treated with ALAAgNP6. The two-sample *t*-test between water and ALA groups and between water and ALAAg groups - a highly significant *p*-value <0.001 (0.1%). (c) The role of ALA concentration in the UV-vis spectra and (d) chlorophyll production compared with ALAAgFeNP10. SEM of a Plant treated with ALAAgNP7. The difference between the average of the ALA and ALAAgNP8 populations is not big enough to be statistically significant; between ALA and other groups, *p* < 0.05 and statistically significant.

Fig. 6a shows UV-vis spectra of ALAAgNPs with increased AgNO_3_ concentration. A broadening in the SPR band with an increase in AgNO_3_ concentration indicates the formation of agglomerates (SEM image for ALAAgNP6). The role of AgNPs in chlorophyll synthesis/destruction in plants is demonstrated in Fig. 6b. This figure shows the chlorophyll fluorescence intensity of plant-treated nanoparticles in the function of silver concentration. The result indicated that the reduction of chlorophyll contents increased with the increased concentrations of silver in NPs, indicating inhibition of plant growth. The toxicity of AgNPs may result from their particles directly or indirectly from the release of Ag^+^ ions.^49^

The UV-vis obtained for three different ALA concentrations used in the synthesis of ALAAgNPs is shown in Fig. 6c. The toxicity of ALA for the plant is shown in Fig. 6d. The result indicated that the reduction of chlorophyll contents increased with the increased concentrations of ALA in NPs. In Fig. 6d, it is also observed that ALAAgFeNP10 chlorophyll fluorescence intensity was increased compared to ALA-treated plants.

### Antimicrobial test

**Fig. 7:**
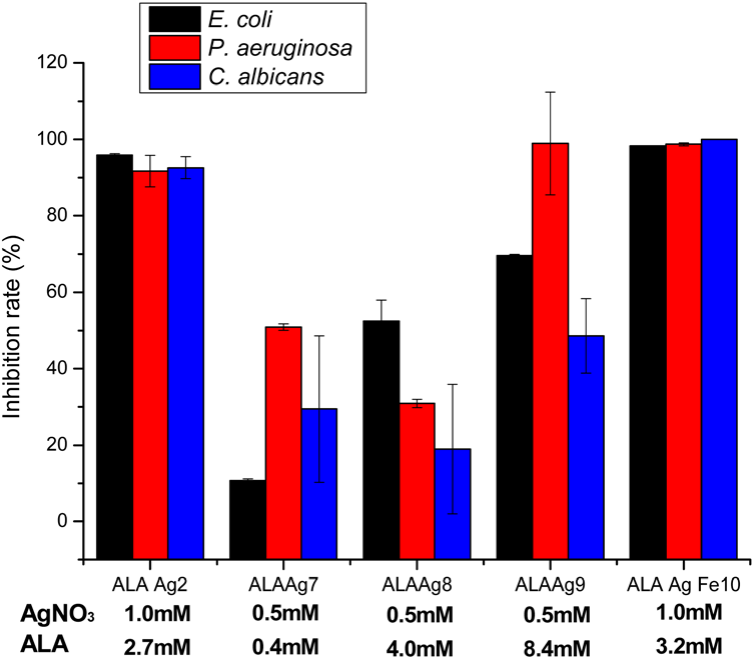
The antimicrobial activity of ALAAgNP (2, 7, 8, and 9) and ALAAgFeNP10 nanoparticles against *Escherichia coli* ATCC 25922, *Pseudomonas aeruginosa* ATCC 27853, and *Candida albicans* ATCC 10231. No statistical difference was obtained between groups ALAAgNP2 and ALAAgFeNP10. *p*-value <0.05 (except for *C. albicans*), indicates strong evidence of a difference between groups ALAAgNP7, ALAAgNP8, and ALAAgNP 9.

The antimicrobial activities of ALAAgNPs and ALAAgFeNPs were tested using the broth microdilution assay, which provides quantitative data on inhibition efficacy. The results are presented in Fig. 7. The results demonstrated that studied microorganisms (*Escherichia coli* ATCC 25922, *Pseudomonas aeruginosa* ATCC 27853, and *Candida albicans* ATCC 10231) showed high inhibition percentual (>90%) when exposed to ALAAgNP2 (high silver concentration) and ALAgFeNP10 nanoparticles. Moreover, the concentration of ALA in NPs influences the antimicrobial activity of ALANPs.

## Discussion

Through the photoreduction process was possible to synthesize ALAAgNPs from an aqueous solution containing ALA and AgNO_3_ without introducing stabilizers or surfactants. This process rapidly transferred electrons to the Ag^+^ cations reducing them into Ag^0^. ALA molecules surrounded silver clusters protecting and stabilizing the nanoparticles. In the case of ALAAgFeNPs, when iron powder (Fe_3_O_4_ magnetite) was added to the solution, the iron underwent oxidation in contact with water, being transformed into Fe^0^ and surrounded by ALA molecules. Produced ALAAgNPs and ALAAgFeNPs were spherical and very stable.

The release of singlet oxygen by irradiation around 590 nm (Fig. 3) was observed for ALAAgFeNPs. This result indicates the potential of a direct generation of singlet oxygen by pulverization of ALAAgFeNPs in plants and soil under sun exposure before their internalization. In the case of ALAAgNPs, sun exposure could induce an energy or electron transfer from the triplet state of PpIX (present in plant cells due to ALA metabolism) to the molecular oxygen, leading to the formation of ROS responsible for killing microorganisms.

ALANPs interactions with terrestrial and aquatic environments, as well as human exposure and toxicity, are of significant concern. The nanoparticle accumulation in the human body and plants may cause severe problems at long-term interactions and high concentrations.^35,50–52^

The toxicity of ALANPs in *E. densa* was studied by cell morphology and chlorophyll quantification to explore ALANPs uptake, accumulation, degradation, and chlorophyll synthesis. *E. densa* was chosen due to its fast-growing and ability to uptake nanoparticles through roots and leaves.^53^

The plant cell wall comprises pores (10–50 nm)^54^ and acts as a natural filter.^40^ Small NPs transit through the pores and enter the plasma membrane, whereas large-sized NPs are filtered out. The interaction with NPs creates new and large-sized pores in the cell wall, increasing the cell internalization efficiency. Other possibilities of internalization are through endocytic processes, ion channels, or transport carrier proteins.^40^ The permeability of the cell membrane is affected by the presence of AgNPs.^55,56^ Larger NPs can be clogged in the root or leaves tissues, but smaller ones may be transported to root and leaf tissues with increasing concentrations of NPs.^57^

Results shown in Fig. 4a indicate the presence of ALAAgNPs in the cell wall and ALAAgFeNPs in vacuoles. Higher silver and iron concentrations damaged and disrupted plant cell and chloroplast structures. In these cases, the leaves acquired a gray color for AgNPs-treated samples and red for AgFeNPs-treated samples.

Fig. 4b shows that ALAAgNPs accumulated on the leaf surface. Results indicated that iron nanoparticles in ALAAgFeNPs seem to facilitate nanoparticle internalization.

After entry into cells and chloroplasts (Fig. 4a), the presence of nanoparticles induces the formation of intracellular reactive oxygen species (ROS) such as singlet oxygen, superoxide, and hydrogen peroxide.^58^ The increased generation of hydrogen peroxide affects the growth and development of plants and kills cells. Increased hydrogen peroxide concentration also promotes nanoparticle disruption, and ALA, iron, and Ag^+^ ions are released, creating biological alterations.^59^

It is known that the exogenous application of ALA in plants can promote photosynthesis.^60^ ALA induces the accumulation of chlorophyll.^3,10,61^Fig. 5 indicates nanoparticles induce chlorophyll accumulation due to ALA delivery inside plant cells. At the same time, the oxidative stress promoted by NPs and metal ions led to various toxic impacts and affected the gene expressions and the destruction of DNA. Silver species have a tolerance limit up to a particular concentration above which damage to cells starts, and the chlorophyll synthesis is interrupted (Fig. 6b).^62^

The results shown in Fig. 6d corroborate the results shown in the literature that high ALA concentration causes growth suppression, evidenced by the decrease in chlorophyll fluorescence.^10,11,63^

Antimicrobial tests of ALA, ALAAgNPs, and ALAAgFeNPs presented in Fig. 7 show that the rate between ALA and silver nitrate concentrations in ALAAgNPs synthesis highly influences the antimicrobial activity of AgNPs. 1 mM of silver nitrate concentration promoted higher growth inhibition (ALAAgNP2 and ALAAgFeNP10) than 0.5 mM. Although it was not observed statistical difference between ALAAgNP2 and ALAAgFeNP10 is observed that ALAAgFeNP10 was highly effective in killing microorganisms. Fixing AgNO_3_ concentration in synthesis and varying ALA concentration from 0.4 to 8.4 mM was observed an increase in the growth inhibition rate for *E. coli*, *P. aeruginosa*, and *C. albicans*. However, no statistical significance was obtained for *C. albicans*.

The penetration of AgNPs in Gram-negative bacteria such as *E. coli* and *P. aeruginosa* depend on their size.^64^ The change in expression of heat shock proteins due to the impact of AgNPs^65^ disrupts the bacterial membrane, and the entrance of the smaller particles in the cell membrane becomes easy. In our studies, iron in ALAAgFeNPs alters the NPs size, increases cell penetration, and consequently increases antimicrobial activity.

The inhibition rate of *C. albicans* cells reached ∼99% with ALAAgFeNPs, which undoubtedly documented the effectiveness of the inhibition method. This result is even better than other authors' results in PDT studies.^46^

The obtained results here only demonstrate the antimicrobial activity of ALANPs, but pathogens responsible for losses in agricultural productivity should be tested.

This work indicated important properties of ALANPs depending on the silver and ALA concentrations that allow their use in agriculture in applications such as seed priming, plant-growth promotor, and nanopesticide. (1) ALA is a natural metabolite existing in all living cells and possesses low toxicity; (2) ALANPs exhibit high antimicrobial activity; (3) ALANPs promoted plant growth; (4) ALANPs have the potential to exhibit photodynamic herbicidal properties under solar illumination on the surface of plants (AgFeNPs) and when uptake by the plants (AgNPs).

## Conclusions

ALAAgNps and ALAAgFeNPs were successfully synthesized by the photoreduction method. The ALAANPs presented spherical shapes, sizes of ∼30 nm and 30 and 12 nm, and zeta potential ∼−37 mV and ∼31 mV for ALAAg and ALAAgFeNPs, respectively. *E. densa* treated with ALANPs ([Ag] ∼0.5 mM and [ALA] ∼0.4 mM) presented higher chlorophyll content compared with water or ALA-treated plant and no signal of toxicity. ALAAgFeNPs ([Ag] ∼0.5 mmol and [ALA] ∼3 mM and [Fe] ∼0.3 mmol) promoted plant growth and presented photodynamic activity when illuminated at ∼590 nm. Antimicrobial tests indicate that the inhibition rate of studied microorganisms increased with increasing ALA concentration and in the presence of iron in ALANPs synthesis. ALAAgNPs and ALAAgFeNPs could be suitable for applying as environment-friendly agents, limiting the rampant usage of harmful agrochemicals. It also could contribute to overcoming multidrug resistance to pests, enhancing crop yield while reducing microbial attacks.

## Author contributions

LCC designed the study and conducted the data analysis and interpretation of the results. ISL synthesized the ALANPS. SSB, MRF, and DSC performed antimicrobial studies. All authors drafted the manuscript authors to read and approve the final manuscript.

## Conflicts of interest

The authors declare that there is no conflict of interest.

## Supplementary Material
